# Application of Thermally Responsive Elastin-like Polypeptide Fused to a Lactoferrin-derived Peptide for Treatment of Pancreatic Cancer 

**DOI:** 10.3390/molecules14061999

**Published:** 2009-06-04

**Authors:** Iqbal Massodi, Emily Thomas, Drazen Raucher

**Affiliations:** Department of Biochemistry, University of Mississippi Medical Center 2500 N. State Street Jackson, MS 39216, USA; E-mail: imassodi@biochem.umsmed.edu (I.M.), ehthomas@biochem.umsmed.edu (E.T.)

**Keywords:** Elastin-like polypeptide, L12, hyperthermia, thermal targeting, drug delivery, pancreatic cancer

## Abstract

A well characterized, peptide derivative of bovine lactoferrin, L12, has been shown to possess anticancer properties in multiple cell lines. However, adverse side effects in normal tissues and poor plasma kinetics that hinder the clinical effectiveness of current chemotherapeutics also deter the potential for effective delivery of this L12 peptide. To overcome these limitations, we have developed an Elastin-like polypeptide (ELP) carrier that has the potential to thermally target therapeutic peptides and chemotherapeutics to a tumor site. The coding sequence of ELP was modified with the L12 peptide at the C-terminus and a membrane transduction domain derived from the HIV-1 Tat protein at the N-terminus (Tat-ELP-L12). The thermally responsive Tat-ELP1-L12 is soluble in aqueous solutions at 37°C but aggregates near 41°C, which makes Tat-ELP1-L12 ideal for targeting to solid tumors on application of focused hyperthermia. We observed that under hyperthermia conditions at 42°C, Tat-ELP1-L12 mediated cytotoxicity in MIA PaCa-2 pancreatic adenocarcinoma cells was enhanced by nearly thirty-fold. We investigated the mechanisms of cell death and found evidence of mitochondrial membrane depolarization and caspase activation, which are characteristic of apoptosis, as well as, increased membrane permeability, as shown by LDH release. These results suggest that Tat-ELP1-L12 possesses cytotoxic properties to cancer cells *in vitro* and may have the potential to provide an effective vehicle to thermally target solid tumors.

## 1. Introduction

Systemic delivery of chemotherapeutics at doses required to kill cancer cells causes many adverse side effects in normal tissues. Therefore, the necessity to develop therapies that are specific to tumor cells and/or are targeted to the tumor site is apparent. Various delivery systems are being optimized for targeted drug delivery, including liposomes [1,2], microspheres and nanospheres [3], and macromolecular synthetic polymers [4,5,6] among others. Macromolecular carriers, in particular, are appealing, because they enhance drug stability, exploit the enhanced permeability and retention effect (EPR) of tumor vasculature [7,8,9,10], and can be actively targeted to the tumor site [11,12]. In this study, we demonstrated that a thermally responsive macromolecular carrier can deliver a lactoferrin-based lytic peptide, L12, and inhibit cancer cell growth. Previous studies of bovine lactoferrin and its pepsin-digested moiety, lactoferricin, confirm their ability to select for and induce apoptosis in cancer cells [13,14,15,16,17]. In order to increase its anti-tumor activity, the amino acid sequence of lactoferrin was shortened to residues 14-31 of the alpha helical region and was altered to increase the overall positive charge of the molecule, as described by Yang *et al*. [14]. This L12 peptide has been shown to inhibit growth of numerous tumor cell lines, including murine fibrosarcoma (MethA), human colorectal adenocarcinoma (HT-29), and the human mammary carcinoma cell line MT1 [13,14]. 

Even though lytic peptides are increasingly being tested for the treatment of different cancers, they have limited cellular access and poor pharmacokinetic parameters, rendering them ineffective *in vivo* [13,14,18,19,20]. As shown by previous studies, cell permeability and pharmacokinetic characteristics of therapeutic peptides may be dramatically improved by conjugating these peptides to cell penetrating peptides and macromolecular carriers [6,21,22]. Therefore to create an effective carrier system to target and deliver the lytic peptide L12 to the tumor site, we conjugated L12 to elastin-like polypeptide (ELP) and to the cell penetrating peptide (CPP), Tat. ELP is a biopolymer derived from the structural motif found in mammalian elastin protein and has a sequence-dependent transition temperature that can be utilitized to thermally target the drug delivery vector to solid tumors [23,24]. ELP is composed of a pentapeptide repeat, Val-Pro-Gly-Xaa-Gly, where Xaa can be any amino acid except proline. The guest residue and number of repeats can be modified to alter the phase transition temperature (T_t_) of the polypeptide, evidenced in the difference in the transition temperatures of ELP1 and ELP2. At temperatures higher than T_t_, ELP will undergo a reversible phase transition, form insoluble aggregates, and be taken up into the cell en masse. By taking advantage of this property of ELP, previous *in-vivo* studies show a 2-fold increase in ELP accumulation in heated tumors, as compared to non-heated tumors after systemic administration [12,22]. Furthermore, hyperthermia itself has been shown to increase tumor drug delivery, because it selectively increases the permeability and perfusion of tumor vasculature more than normal vasculature [25,26,27]. Therefore ELP-based delivery of chemotherapeutics combines the advantages of passive targeting, due to the EPR of tumor vasculature, and active targeting, due to the accumulation of thermally responsive ELP when hyperthermia is applied.

Inefficient translocation of these macromolecular carriers across the cell membrane often hinders drug delivery. One way to overcome this problem is to conjugate a CPP to the ELP carrier. CPPs are short, 10-30 amino acid peptides that aid in the intracellular transport of various cargos [21,28,29,30]. We have shown that different CPPs (Antp, Tat, and MTS) were able to effectively translocate ELP across the cell membrane [31]. In previous studies the transduction domain from HIV-1 Tat increased the internalization of thermally responsive ELP1 and ELP1-GFLG-Dox nearly 10-fold upon application of hyperthermia [11,32,33]. In the current study, ELP was modified at the *N*- and *C*-termini by the addition of Tat and L12, respectively. This thermally responsive Tat-ELP1-L12 polypeptide inhibited *in vitro* proliferation of MIA PaCa-2, Panc-1, MCF-7, and SKOV-3 cells in combination with hyperthermia. The human pancreatic adenocarcinoma cell line MIA PaCa-2 was especially sensitive to Tat-ELP1-L12 treatment, and one hour application of the polypeptide under hyperthermia conditions resulted in 70-90% inhibition of cell proliferation. We investigated the mechanism of cell growth inhibition in combination with hyperthermia (42 °C) and found evidence of necrosis--cell membrane disruption followed by the release of cytoplasmic lactate dehydrogenase (LDH), and apoptosis--mitochondrial membrane depolarization and caspase activation). These results suggest that thermally-activated Tat-ELP1-L12 can be selectively targeted to and induce apoptosis and necrosis in cancer cells at the tumor site.

## 2. Results and Discussion

### 2.1. Design and thermal properties of Tat-ELP-L12

The polypeptide carrier used in this study is based on Elastin-like polypeptide (ELP), a thermally responsive polypeptide that undergoes an inverse temperature phase transition when the temperature is raised above its characteristic T_t_ [24]. The phase transition of ELP is associated with the formation of ELP aggregates that bind and penetrate the cell membrane, thus increasing the delivery load of the drug conjugate. ELP1-based polypeptides have a MW of 59.2 kDa, are composed of 150 pentapeptide repeats with a guest residue ratio of 5:3:2, Val:Gly:Ala, and were designed to undergo a phase transition around 41 °C, slightly above physiological temperature [34]. The ELP2-based polypeptides have a MW of 61.1 kDa, are composed of 160 pentapeptide repeats with a guest residue ratio of 1:7:8, Val:Gly:Ala, and have a characteristic T_t_ of 67 °C [34]. In contrast to ELP1, ELP2 drug conjugates do not undergo a phase transition at the hyperthermia temperature (42 °C) and thus do not form aggregates in solution.

We altered the coding sequence of ELP by adding the Tat CPP to its *N*-terminus and a lytic peptide to its *C*-terminus. Tat has been shown to increase the uptake of ELP1 nearly 8-fold when combined with hyperthermia [32]. Also, a previous study demonstrated the potential for ELP to deliver a therapeutic peptide. An ELP-fused c-Myc inhibitory peptide prevented the proliferation of MCF-7 breast cancer cells [35]. In the present study, we conjugated a lytic peptide that has been shown to exhibit anti-cancer activity against different cancer cell lines to ELP [13,14]. This peptide, L12 (14-31), was derived from the *N*-terminal, α–helical region of bovine lactoferrin, and was added to the *C*-terminus of the ELP coding region. 

**Figure 1:**
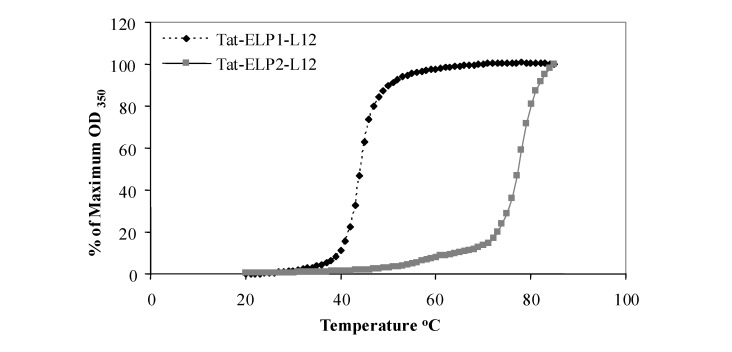
Thermal properties of Tat-ELP-L12 polypeptide.

The coding sequence of the ELP conjugate may alter its transition temperature [36]. Therefore to demonstrate that once Tat and L12 were added to the ELP sequence, the conjugate maintained its characteristic transition temperature, we measured the T_t_ of Tat-ELP1-L12 and Tat-ELP2-L12. Figure 1 shows the turbidity of 20 μM Tat-ELP1-L12 and Tat-ELP2-L12 polypeptide solutions as a function of temperature. Solutions of both polypeptides were clear below the physiological temperature, but as the temperature increased above 41 °C, there was a sharp increase in the turbidity of Tat-ELP1-L12, which was reflected by the steep transition curve. In contrast, the aggregation of Tat-ELP2-L12, which serves as a control for the effect of hyperthermia, occured near 75 °C. The transition of each polypeptide was reversible (data not shown), which is consistent with previously reported thermal properties of ELPs [12]. 

The turbidity profiles of 20 μM of Tat-ELP1-L12 and Tat-ELP2-L12 in PBS were obtained by heating at a rate of 1 °C/min. The temperature at which the polypeptide solutions reached 50% turbidity is defined as the transition temperature Tt.

### 2.2. Effect of polypeptides on cell proliferation

**Figure 2:**
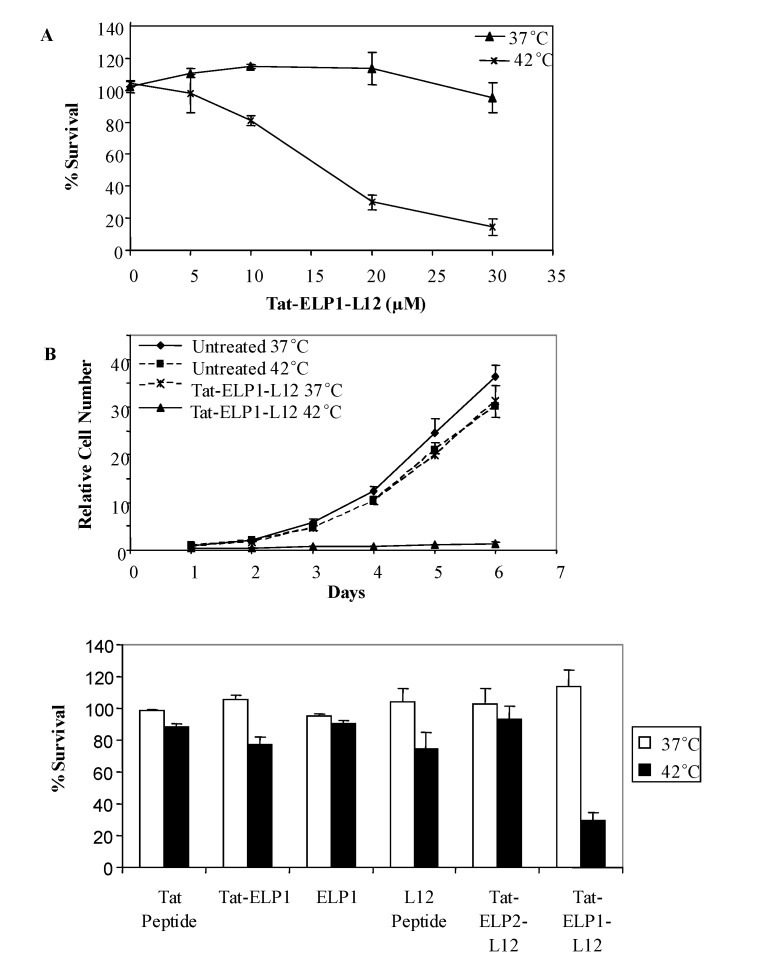
Temperature dependent effect of polypeptides on Mia-Paca-2 cells.

In order to determine the effect of these polypeptides on cell proliferation, MIA PaCa-2 cells were treated with Tat-ELP1-L12 in a concentration dependent manner and immediately placed in an incubator set at 37 °C or 42 °C for 1 hour. After the 1 h exposure, polypeptides were removed and fresh media was replaced, and the cells were allowed to grow at 37°C. As shown in Figure 2A, treatment with Tat-ELP1-L12 at 42°C resulted in a 70-90% inhibition of cell proliferation between 20-30 μM concentrations, as measured by a colorimetric MTS assay. No effect was observed on the cell proliferation with Tat-ELP1-L12 at 37 °C. These results are consistent with previous studies where hyperthermia augmented the anti-proliferative effect of Pen-ELP1-H1 and Tat-ELP1-GFLG-Dox [11,37,38]. The enhanced inhibition of cell proliferation was reportedly caused by the hyperthermia-induced phase transition of the ELP1 polypeptide, and not by hyperthermia itself. To confirm that actual growth inhibition and not metabolic changes decreased the perceived cell survival, we measured the growth rate of MIA PaCa-2 cells. Untreated cells at 37 °C or 42 °C doubled five times in six days, increasing the number of cells by 30-35 fold. Cells treated with Tat-ELP1-L12 for 1 h at 37 °C showed a similar growth rate as untreated cells; whereas cells treated with Tat-ELP1-L12 at 42 °C did not multiply over the six day period (Figure 2B). Thus Tat-ELP1-L12 treatment in combination with hyperthermia decreased cell proliferation by 30-fold, as compared to untreated cells. Therefore, these results suggest that the mechanism for inhibition of cell growth is due to the cellular internalization of high load ELP1 aggregates, during the 1 hour drug and hyperthermia treatment. 

Cell proliferation measured after treatment with different polypeptides for 1 h at respective temperatures. (A) Effect of Tat-ELP1-L12 treatment on cell proliferation as measured by MTS assay after three days. (B) Growth curves generated by daily cell counts after treatment with 20 μM of the Tat-ELP1-L12 at different temperatures. Daily counts are expressed relative to the counts on day 1, and the data represents an average of 3-5 experiments. Bars- ± SE. (C) Cell proliferation after treatment with 20 μM controls. Results are represented as mean ± SE of 3-5 independent experiments.

In order to parse the effects of the individual components of the drug conjugate, we treated MIA PaCa-2 cells with 20 μM of the Tat peptide, Tat-ELP1, ELP1, L12 peptide or thermally unresponsive Tat-ELP2-L12 (Table 1). None of these peptides, including Tat-ELP1-L12, significantly reduced cell proliferation at 37 °C (Figure 2C). Importantly, Tat-ELP2-L12 did not induce cell death at hyperthermia temperatures, which confirms that the large inhibition seen with Tat-ELP1-L12 at 42 ºC is due to its aggregation, not to nonspecific effects of hyperthermia. The lack of growth inhibition with ELP2 conjugates and with Tat-ELP1-L12 at 37 ºC suggests not only that these conjugates are inefficiently taken up into cells, but also that Tat-ELP1-L12 can be targeted to the tumor site, specifically reducing cancer cell growth, while minimizing systemic toxicity where hyperthermia is not applied.

**Table 1:** Polypeptides used in cell proliferation experiments. ELP is a genetically engineered construct, whose sequence can be modified to include the Tat peptide at the *N*-terminus and the lytic peptide, L12, at the *C*-terminus. Both, Tat and L12, are underlined; the intervening sequences are spacer sequences. The following peptides were used to parse the cytotoxic effect of the individual components in the drug conjugate.

Polypeptide Name	Polypeptide Sequence	Molecular Weight (kDa)
ELP1	SKGPG-(VPGXG)_150_^†^-WP	59.2
ELP2	SKGPG-(VPGXG)_160_^‡^-WP	60.1
Tat-ELP1	YGRKKRRQRRRGGPG-(VPGXG)_150_^†^-WPGSGGC	61.3
Tat-ELP2	YGRKKRRQRRRGGPG-(VPGXG)_160_^‡^-WPGSGGC	62.8
Tat-ELP1-L12	YGRKKRRQRRRGGPG-(VPGXG)_150_^†^	63.5
	WPGSGGPAWRKAFRWAKRMLKKAA	
Tat-ELP2-L12	YGRKKRRQRRRGGPG-(VPGXG)_160_^‡^-WPGSG	64.9
	GPAWRKAFRWAKRMLKKAA	
Tat	YGRKKRRQRRR	1.6
L12	GPAWRKAFRWAKRMLKKAA	2.3

† X represents the amino acids V, G, or A in a 5:3:2 ratio; ‡ X represents the amino acids V, G, or A in a 1:7:8 ratio; * Not applicable

When heated to 42 °C for one hour, however, Tat-ELP1 and the L12 peptide did prevent cell proliferation. Even though Tat alone has no significant effect, we have previously reported the mild cytotoxic effect of Tat-ELP1 [32]; however, Tat-ELP1-L12 showed a marked decrease in cell survival, as compared to Tat-ELP1 alone, and in future studies, an alternative CPP, like SynB1, that is not cytotoxic when conjugated to ELP may be used (data not shown). Under the assay conditions used here, we found that at physiological temperatures, 1 h exposure of cells to the L12 peptide had no effect, but at 42 °C, L12 inhibited cell growth by about 25%. In previous studies, the cytotoxic effect of L12 was characterized after cells had been incubated with the peptide for at least four hours at 37 °C [13,14]. Therefore, these results suggest that at higher temperatures, L12 is more likely to penetrate the cell; however, in combination with the Tat and ELP1, the translocation efficiency and the delivery load both are enhanced and vastly improve the intracellular delivery of L12. Overall, the additive effect of treatment with Tat-ELP1 and L12 did not account for the 70% decrease in cell survival after treatment with 20 μM of Tat-ELP1-L12 in combination with hyperthermia. These results suggest that in combination with hyperthermia, Tat-ELP1-L12 is not only an effective inhibitor of cancer cell growth, but its inhibition can be thermally targeted, as a significant portion of its toxicity can be attributed to the ELP1-mediated phase transition.

### 2.3. Measurement of Lactate Dehydrogenase (LDH) release

Cationic, amphipathic peptides often disrupt membrane integrity by forming pores [39,40]. In an attempt to determine if treatment with Tat-ELP1-L12 resulted in membrane permeabilization, we measured the release of the cytoplasmic enzyme lactate dehydrogenase (LDH). LDH is an oxidoreductase which catalyzes the interconversion of lactate and pyruvate. LDH is often measured to evaluate the presence of tissue or cell damage and cell death [41,42] and can be used to gage membrane permeabilization *in-vitro*. 

**Figure 3:**
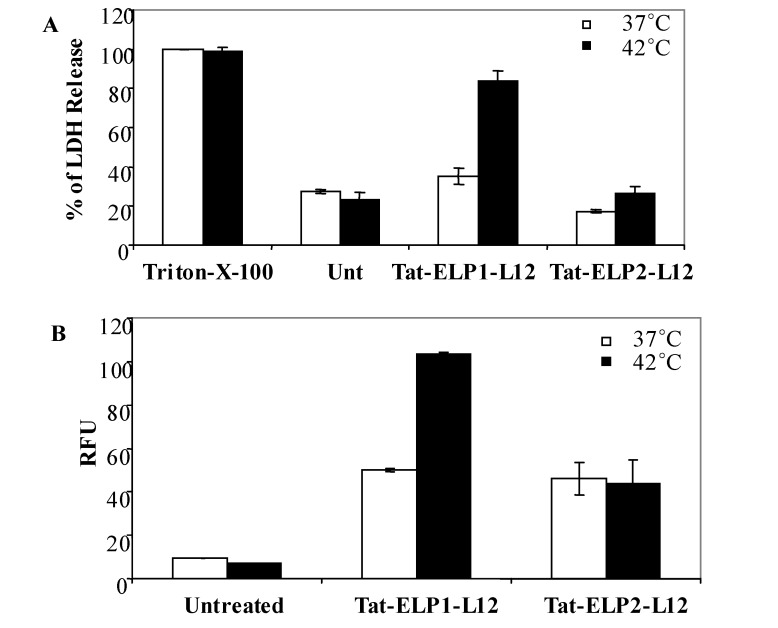
Effect of Tat-ELP1-L12 treatment on membrane permeability.

(A) Measurement of lactate dehydrogenase release. Cells were treated with 20 μM of Tat-ELP1-L12 or Tat-ELP2-L12 at 37 °C or 42 °C for 1 h. The release of LDH was determined by the non-radioactive colorimetric LDH kit. The absorption maximum at 492 nm was measured and plotted as a percentage of 0.1% Triton X-100 induced LDH release. Results are represented as mean ± SE of 3-5 independent experiments. (B) Measurement of FITC-Dextran uptake. Cells were incubated with 20 μM of Tat-ELP1-L12 and 80 μM FD-4.4 at 37 °C or 42 °C for 1 h. The media was then replaced with FD-4.4 containing media and the cells were incubated for additional 3 h. Cells were later harvested and run on flow cytometer. Results are represented as mean ± SEM of 3-5 independent experiments.

In order to measure LDH release after administration of Tat-ELP1-L12 and hyperthermia in MIA PACA-2 cells, we performed an LDH release assay immediately after the 1 h treatment. Cells were treated with 20 μM Tat-ELP1-L12, Tat-ELP2-L12, and 0.1% Triton-X-100 for 1 h at 37 ºC or 42 ºC and analyzed for LDH release in the culture supernatant. The data represents the percentage of LDH released, with the 0.1% Triton-X-100 signal being the positive control (Figure 3A). Tat-ELP1-L12 treatment at 42 °C caused a 2.5 fold increase in LDH release, as compared to Tat-ELP1-L12 treatment at 37 °C, and a 4 fold increase, as compared to spontaneous release in untreated cell and in Tat-ELP2-L12 treated cells at 37 °C and 42 °C [43]. The elevated LDH release in response to treatment with Tat-ELP1-L12 at 42 °C indicates that the phase transition of Tat-ELP1-L12 mediates the enhancement of the cell membrane permeability that leads to LDH leakage. 

### 2.4. Measurement of FITC-Dextran uptake

Lactoferrin based peptides have previously been shown to cause membrane damage in Jurkat T-leukemia cells, which was assessed by monitoring the release of ^15^Cr-labeled intracellular proteins [15]. To further investigate membrane integrity, leakage, and pore formation due to Tat-ELP1-L12 and hyperthermia treatment, we measured the cellular uptake of FITC-Dextran (FD-4.4) by flow cytometry. FD-4.4 (where 4.4 is the molecular weight in kDa) is a high molecular weight compound that cannot permeate intact cell membranes. Co-incubation of cells with polypeptides and FD allowed us to estimate the uptake of FD through the cell membrane and therefore assess the degree of membrane permeability in response to treatment. As shown in Figure 3B, Tat-ELP1-L12 at 37 ºC or Tat-ELP2-L12 at both temperatures caused an increase in FD uptake by 5-fold as compared to the untreated cells, and this enhanced uptake was likely due to the membrane permeabilization effects of both Tat and L12. Furthermore, the relative fluorescence intensity units (RFU) of FD were 2-fold higher when incubated with Tat-ELP1-L12 at 42 °C as compared to 37 °C, but no heat effect was observed with Tat-ELP2-L12. This finding indicates that an enhanced FD uptake occurs through pore formation mediated by Tat-ELP1-L12 and is not due to heat itself. Furthermore the relative increase in FD uptake between cells treated with Tat-ELP1-L12 and Tat-ELP2-L12 at 37 °C and 42 °C suggests that the ELP1-based phase transition enhances the uptake and therefore activity of L12 in cancer cells.

### 2.5. Tat-ELP1-L12 also induces apoptotic cell death

Mader *et al*. demonstrated that Lactoferricin B not only causes membrane damages and cell necrosis, but also selectively induces apoptosis in Jurkat T-leukemia cells by the mitochondrial pathway [15]. Therefore to investigate whether Tat-ELP1-L12 also induces apoptosis, we examined the mitochondrial membrane potential in MIA PaCa-2 cells following treatment. Flow cytometry was used to measure the intensity of JC-1 dye emission; whereby predominant red fluorescence indicates the accumulation of JC-1 dye in healthy mitochondria, and the aggregation of the monomeric green form of JC-1 in mitochondria evidences mitochondrial collapse. After 1 h exposure to 20 μM of Tat-ELP1-L12 at 42 °C, 60% of cells had undergone mitochondrial membrane depolarization and collapse.

**Figure 4:**
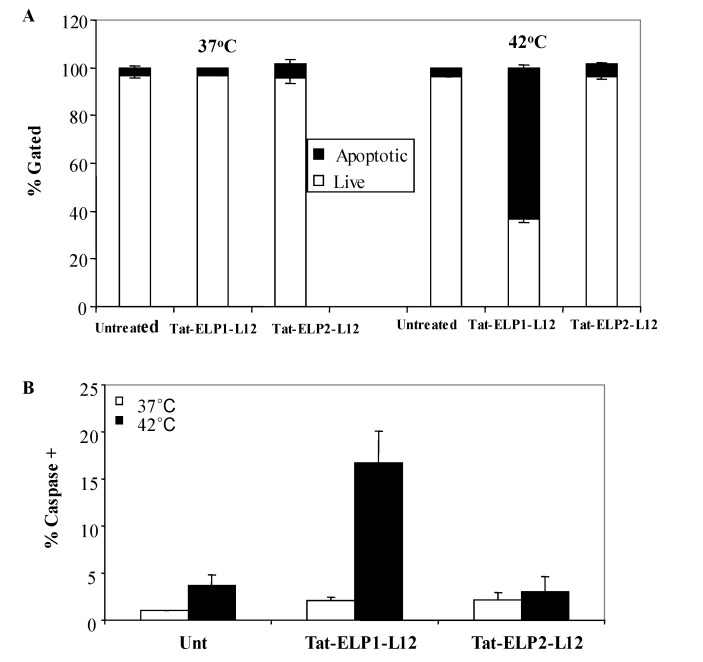
Measurement of Tat-ELP1-L12 induced apoptosis.

Furthermore, we directly measured caspase activation by flow cytometry to evaluate if the mitochondrial destabilization resulted in cytochrome c release and activation of the cell’s intrinsic apoptotic pathway [44,45]. MIA PaCa-2 cells were exposed for 1 h at 37 °C or 42 °C to the indicated polypeptides in Figure 4B. Cells were harvested immediately, incubated with a fluorescent-caspase inhibitor, and analyzed by flow cytometry. As shown in Figure 4B, there was an 8-fold increase in the number of caspase positive cells following treatment with Tat-ELP1-L12 at 42 °C as compared to 37 °C. Hyperthermia treatment alone and treatment with the control polypeptide Tat-ELP2-L12 at both temperatures did not induce caspase activity in MIA PACA-2 cells. These results suggest that cells exposed to Tat-ELP1-L12 and hyperthermia underwent apoptosis by mitochondrial membrane depolarization and caspase activation. These results are confirmed by previous studies that have reported that lactoferricin B kills leukemia and neuroblastoma cells through mitochondrial depolarization and caspase activation, in addition to immediate permeabilization of the cell membrane upon entry [15,17].

(A) Measurement of mitochondrial membrane depolarization. Cells were treated with 20 µM of polypeptides at 37 °C or 42 °C for 1 h, harvested 24 h later and incubated with 1x JC-1 dye for 15 min at 37 °C. Cells were then analyzed on a flow cytometer for the change in mitochondrial membrane potential that is indicated by a decrease in red fluorescence intensity (FL2) and an increase in green fluorescence intensity (FL1). (B) Effect of Tat-ELP1-L12 on caspase activation of Mia-Paca-2 cells. Cells were treated with 20 µM of polypeptides at 37 °C or 42 °C for 1 h. Cells were immediately harvested, washed with PBS and incubated with 10 µM of 30x FLICA reagent for 2 h, and analyzed by flow cytometry. The data represents the average of ± SE of three independent experiments plotted as % caspase positive cells normalized to untreated cells.

### 2. 5. Cell proliferation in other cell lines

**Figure 5:**
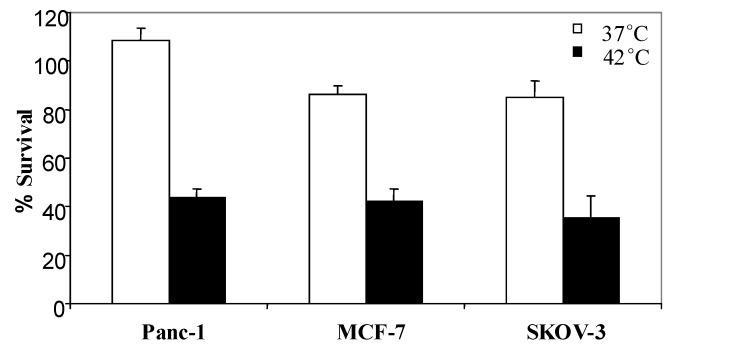
Effect of Tat-ELP1-L12 in different cell lines.

To demonstrate that Tat-ELP1-L12 mediated cytoxicity is not limited to the MIA PaCa-2 cell line, we examined the cytotoxic effect of Tat-ELP1-L12 in Panc-1 (pancreatic cancer), MCF-7 (breast cancer) and SKOV-3 (ovarian cancer) cell lines. Cells exposed to Tat-ELP1-L12 at 42 ºC showed nearly 60-70% inhibition of cell proliferation in all cell lines, as compared to treatment at 37 ºC (Figure 5). These results suggest that the phase-transition mediated cytotoxic effect of Tat-ELP1-L12 is independent of cell type and therefore may be applied to treat many different cancers. 

Cells were treated with 20 µM Tat-ELP1-L12 for 1 h at respective temperatures and the cell proliferation was measured by MTS assay after 3 days.

### 2.6. Measurement of Hemolytic activity

One of the concerns associated with lytic peptides and other drug delivery systems is that they should possess either low or no hemolytic activity. The L12 peptide and Tat-ELP1 polypeptide have been reported to show no hemolytic activity [13,32]. 

**Figure 6:**
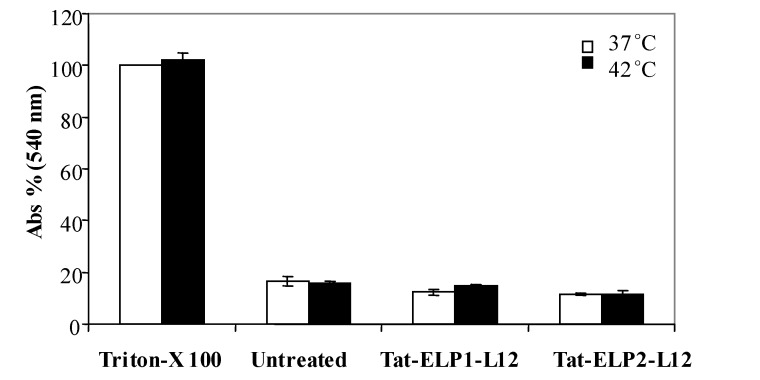
Hemolytic activity of Tat-ELP1-L12.

Murine RBCs were incubated with 30 µM Tat-ELP1-L12 or Tat-ELP2-L12 at 37 °C or 42 °C for 1 h. Absorbance of the supernatant was recorder at 540 nm and plotted as a percentage of 0.1% Triton X-100 induced hemoglobin leakage. Results are expressed as mean ± SE of 3-5 independent experiments.

Here we examined the hemolytic activity of the chimeric polypeptides Tat-ELP1-L12 and Tat-ELP2-L12 at 37 °C or 42 °C in murine erythrocytes (Figure 6). Murine erythrocytes in PBS were incubated with the polypeptides, and hemolysis was assessed by monitoring the release of hemoglobin spectrophotometrically. Figure 6 shows no increase in the absorbance of samples treated with Tat-ELP1-L12 or Tat-ELP2-L12 as compared to untreated samples, indicating no hemolytic effect at either 37 °C or 42 °C. 

## 3. Experimental

### 3.1. Design of Constructs

The pUC19-ELP1 and pUC19-ELP2 were synthesized as described previously [11,32,36]. The ELP coding sequence was modified by the addition of the Tat (MYGRKKRRQRRR) coding sequence to the *N*-terminus and the L12 (GPAWRKAFRWAKRMLKKAA) coding sequence to the *C*-terminus. Sequences of all synthesized constructs were confirmed by DNA sequencing.

### 3.2. Polypeptide purification

pET25b+ expression vectors containing the desired constructs were transformed into *E. coli* BLR (DE3) (Novagen, Madison, WI, USA) for protein hyperexpression [46]. Cells were then harvested by centrifugation, and the protein was extracted as described previously [31,37].

### 3.3. Characterization of transition temperature

The temperature-induced aggregation of the proteins was characterized by monitoring absorbance at 350 nm as a function of temperature. Solutions of Tat-ELP1-L12 and Tat-ELP2-L12 containing 20 μM protein in PBS were heated or cooled at a constant rate of 1°C/min in a temperature-controlled multicell holder in a UV-visible spectrophotometer (Cary 100, Varian Instruments, Cary, NC, USA). The T_t_ was defined as the temperature at which the *A*_350_ reached 50% of the maximum turbidity. 

### 3.4. Cell culture and polypeptide treatment

Pancreatic cancer, MIA PaCa-2 and Panc-1, (ATCC, Manassas, VA, USA) cells were grown in DMEM, SKOV-3 in McCoy’s-5a media (Invitrogen, Carlsbad, CA, USA) supplemented with 10% fetal bovine serum (Sigma, St. Louis, MO, USA), 100 units/ml penicillin, 100μg/ml streptomycin, and 25 μg/mL amphotericin B (Invitrogen). MCF-7 breast cancer cells were grown in MEM supplemented with 10% fetal bovine serum, 1 mmol/L sodium pyruvate, Basal Medium Eagle amino acids, 5 μg/mL insulin (Sigma), 100 units/mL penicillin, 100 μg/mL streptomycin, and 25 μg/mL amphotericin B. Cultures were maintained at 37 °C in a humidified atmosphere + 5% CO_2_. For experiments, cells were removed from tissue culture flasks by brief treatment with 0.05% v/v trypsin-EDTA (Invitrogen) and plated in 6 well plates (500,000 cells/ well) for flow cytometry and in 96 well plates for proliferation. Twenty-four hours after plating, cells were treated with media containing the indicated concentration of polypeptides for 1 h, rinsed, and replaced with fresh media. 

### 3.5. Cell proliferation

MIA PaCa-2 cells were plated in 96 well plates (5,000/ well), allowed to attach overnight, and treated with Tac-ELP1-L12, Tat-ELP2-L12, Tat-ELP1, or L12 peptide at various concentrations. The 96 well plates were immediately placed in incubators that were set to either 37 °C or 42 °C, and the plates were removed after 1 hour. Cells were washed and fresh media was replaced. Cell proliferation was measured using the CellTiter 96^®^ AQ_ueous_ Assay kit (Promega). The effect of polypeptides on the proliferation rate of MIA PaCa-2 cells was determined by plating in a 24 well plate (10,000 cells/ well). Cells were treated with Tat-ELP1-L12 (20 μM) for 1 h at 37 or 42°C, and the cell counts were made daily using a Coulter counter (Coulter, Fullerton, CA, USA). Panc-1 and MCF-7 cells (8,000 cells/ well), and SKOV-3 cells (6,000 cells/ well) were plated as described above and treated with 20 μM Tat-ELP1-L12 for 1 h at 37 or 42 °C. Cell proliferation was measured using the MTS assay and the data represents average of three to five experiments.

### 3.6. Lactate dehydrogenase relase (LDH) assay

MIA PaCa-2 cells were plated in a 96 well plate (15,000/ well). Cells were incubated with 20 μM Tat-ELP1-L12 or Tat-ELP2-L12 at 37 or 42 for 1 h. The amount of LDH released was quantified using a CytoTox 96 Non-Radioactive Cytotoxic Assay kit (Promega). For a positive control, cells were treated with 0.1% Triton X-100 (Sigma) lysis solution. The supernatant was transferred to new wells and treated with a dye solution according to the CytoTox assay protocol. LDH activity was quantified by measuring the absorbance at 490 nm using a plate reader (Tecan Systems Inc. San Jose, CA, USA). All the experiments were repeated three to five times. 

### 3.7. FITC-Dextran uptake assay

MIA PaCa-2 cells were plated in 6 well plates (500,000/ well). Cells were treated with a mixture 20 μM Tat-ELP1-L12 or Tat-ELP2-L12 and 80 μM FD-4.4 (where 4.4 is M.W in kDa) at 37 or 42 ºC for 1 h. After 1 h treatment, the media was replaced with media containing only FD and incubated at 37°C for three additional hours. Cells were later harvested with trypsin, washed and resuspended in PBS and analyzed using a Cytomics FC 500 flow cytometer and CPX software (Beckman Coulter, Fullerton, CA, USA). Dot plots of forward vs. side scatter were used to exclude cell debris from analysis. 

### 3.8. Caspase detection assay

MIA PaCa-2 cells were plated at 500,000 cells/ well in a 6 well plate, grown overnight and treated with 20 μM Tat-ELP1-L12 or Tat-ELP2-L12 at 37 °C or 42 °C for 1 h. Fresh media was added to cells after 1 h incubation. Cells were then washed with PBS and harvested using trypsin. Caspase activity was detected by staining the cells with 10 μL of 30x carboxyfluorescein FLICA-reagent for 2 h as per the protocol of FLICA-Apoptosis Poly-caspase Detection Kit (Immunochemistry Tech. Bloomington, MN, USA). Cells were rinsed twice with apoptosis wash buffer and analyzed for caspase activation by flow cytometry using a Cytomics FC 500 flow cytometer (n = 5,000 cells). Forward versus side scatter gating was used to eliminate cell debris from the analysis, and the histogram of fluorescein fluorescence (channel FL1) was bimodal with peaks for caspase positive and caspase negative cells. Cells under the high intensity peak were quantified as cells with active caspases, whereas cells with low intensity represented caspase inactive cells. The percentage of caspase positive cells was expressed as an average of at least 3 experiments; error bars, ±SE.

### 3.9. Mitochondrial membrane depolarization assay

Change in the mitochondrial membrane potential was assessed by incubating cells with JC-1 dye (Cell Technology Inc. Mountain View, CA, USA). MIA PaCa-2 cells were plated at 500,000 cells/well in a 6 well plate, grown overnight and treated with 20 μM Tat-ELP1-L12 or Tat-ELP2-L12 at 37°C or 42˚C for 1 h. Fresh media was added to cells after the 1 h treatment and the cells were incubated for additional 24 h. Cells were then washed with PBS and harvested using trypsin. Change in the mitochondrial membrane potential was detected by incubating cells at 37 °C with 1x JC-1 reagent for 15 min as per the protocol and analyzing them using a Cytomics FC 500 flow cytometer (Beckman Coulter). Samples of 10,000 cells were examined on a FL-1 (520 nm) versus FL-2 (570 nm) scatter plot.

### 3.10. Hemolytic activity

Fresh blood was collected from Sprague Dawley rats (Charles River Laboratories Inc., Frederick, Maryland, USA), stored at 4°C in heparinized tubes, and used within 10 days. Blood (1 mL) was centrifuged for 10 min at 1000g, and the pellet was resuspended in PBS to make a 4% cell suspension. Fifty μL of the cell suspension was transferred to tubes containing 950 μL of 30 μM Tat-ELP1-L12, Tat-ELP2-L12 or 950 μl of 0.1% Triton-X-100. After 1 h of incubation at 37 or 42 °C, the vials were centrifuged at 1000g for 2 min, and the amount of released hemoglobin in the supernatant was measured by monitoring the absorbance at 540 nm using a UV-visible spectrometer (UV-1600 Shimadzu). 

## 4. Conclusions

In the present study, we have shown that the ELP-based drug delivery system carrying a lactoferrin-derived peptide L12 has the potential to be thermally targeted to cancer cells and to inhibit their proliferation. By conjugating the lytic peptide L12 to the ELP-based delivery system, we overcome several obstacles that thwart drug development, including poor pharmacokinetics and systemic toxicity. Lactoferrin derivatives, like L12, are potent activators of cell death, as they both induce necrosis by puncturing the plasma membrane and apoptosis by activating intracellular caspase cascades in response to mitochondrial membrane damage. The cytotoxic activity of L12 can be modulated by conjugating this peptide to macromolecular carrier, ELP1, which preferentially aggregates and infiltrates tumor cells upon induction of hyperthermia, as has been shown in this study. Furthermore, we have shown that Tat-ELP1-L12 in combination with heat potently inhibits tumor cell proliferation *in-vitro* and induces cell death, by cell necrosis and apoptosis. This conjugate is a promising candidate for future *in vivo* studies because of its degree of selectivity in inducing cancer cell inhibition under hyperthermic conditions. 
